# Enhancing stroke care in Europe: cost-effectiveness analysis of strategies addressing gaps in acute ischemic stroke care

**DOI:** 10.1093/esj/aakag041

**Published:** 2026-05-19

**Authors:** Bianca de Greef, Ji-Hee Youn, Henri V Bavière, Marjan Hummel, Angelique Balguid, Francesca Pezzella, Hanne Christensen, Wim van Zwam

**Affiliations:** Chief Medical Office, Health Economics and Outcomes Research, Philips, Amsterdam, the Netherlands; Chief Medical Office, Health Economics and Outcomes Research, Philips, Amsterdam, the Netherlands; Chief Medical Office, Health Economics and Outcomes Research, Philips, Amsterdam, the Netherlands; Chief Medical Office, Health Economics and Outcomes Research, Philips, Amsterdam, the Netherlands; Image-Guided Therapy Systems, Philips, Best, the Netherlands; Stroke Unit, Department of Neuroscience, S Camillo Forlanini Hospital, Rome, Italy; Department of Neurology, Copenhagen University Hospital, Bispebjerg, Denmark; Department of Radiology and Nuclear Medicine, Maastricht University Medical Center+, Maastricht, the Netherlands

**Keywords:** acute ischaemic stroke, cost-effectiveness analysis, decision-analytic modelling

## Abstract

**Introduction:**

Acute ischemic stroke (AIS) is a leading cause of disability and mortality worldwide, resulting in a significant economic burden. The European Stroke Action Plan set targets to improve stroke care, but substantial variation persists across countries.

**Patients and methods:**

A decision-analytic model was developed. By comparing baseline performance in stroke care, based on data from 2015 to 2020, with the performance when meeting targets set on incidence, time and access to treatment and prevention, we estimated the potential clinical and economic impact in 7 European countries. The primary outcome was the incremental cost-effectiveness ratio, based on costs and quality-adjusted life-years (QALYs); secondary outcomes included disability (modified Rankin Scale; mRS).

**Results:**

Achieving the targets produced meaningful health gains in all countries, with several scenarios being dominant. Reducing stroke incidence had the greatest overall impact. Among the remaining targets, shorter time to treatment was dominant in France, Italy and the United Kingdom, and consistently reduced disability across countries (0.9%–14.3% point decreases in patients with mRS 3–5). Increasing endovascular treatment rates showed the greatest effect in Sweden and Spain and remained cost-effective elsewhere (€1823–€5331 per QALY gained). Reducing recurrent stroke delivered the largest cost savings in the Netherlands and the United Kingdom (€436 and €483 per patient, respectively) whilst generating QALY gains.

**Conclusion:**

Although reducing stroke incidence yields the greatest overall benefit, optimal strategies differ between countries. Tailored approaches addressing country-specific care gaps will be essential to maximise health gains, reduce disability and improve economic efficiency of stroke care.

## Graphical Abstract

**Graphical Abstract:**
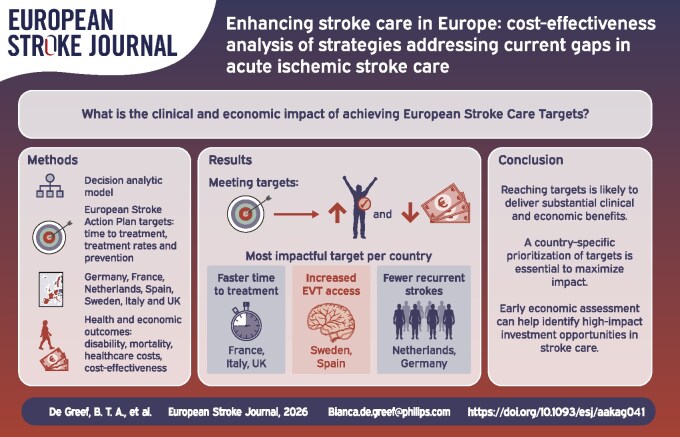

## Introduction

Stroke is a leading cause of mortality and disability worldwide, with ischemic stroke accounting for the majority of cases. ^1,2^ In Europe, the burden of stroke is significant, with approximately 1.8 million new strokes annually in the EU-53^3^ and substantial variability in care delivery across countries.^4,5^ This variability leads to disparities in outcomes, costs and quality of life for patients. Despite advances in acute stroke management, such as the use of intravenous thrombolysis and mechanical thrombectomy, many patients still do not receive timely, optimal care.^4–6^

The European Stroke Action Plan (SAP-E) 2018-2030 set targets to improve stroke care across the continuum, including reducing onset-to-treatment time, increasing treatment rates and addressing (secondary) prevention to reduce stroke incidence and recurrence.^6^ However, the extent to which these targets are being met varies across European countries, with some performing significantly better than others. For instance, countries with well-organised stroke networks and accessible care pathways, such as the Netherlands, demonstrate higher treatment rates and shorter onset-to-treatment times.^4,7^ Conversely, countries with fragmented healthcare systems or regional disparities often face challenges in providing timely and equitable access to care. These disparities mean that patients in some regions experience significantly worse outcomes due to delays in treatment or lack of access to advanced interventions. Identifying and addressing these inequities is critical to formulating evidence-based policies and interventions that improve outcomes while ensuring cost-effectiveness.

The goal of our study was to address identified gaps in stroke care across Europe by evaluating standard practices and assessing their alignment with the SAP-E 2018-2030 targets and by identifying opportunities to optimise health economic outcomes. Through a health economic model, we provided valuable insights into cost-effective strategies that could improve clinical outcomes and optimise resource utilisation. Our findings underscore the importance of targeted interventions to enhance access to care, reduce treatment delays and improve (secondary) prevention strategies, ultimately contributing to increased access to care, better patient outcomes and reduced disparities across countries.

## Patients and methods

### Study design and overview

The primary objective of this study is to quantify the potential clinical and economic impact of achieving the SAP-E targets across different European countries, thereby supporting the prioritisation of country-specific strategies to optimise stroke care.

A model-based economic evaluation was conducted to estimate the value of improving acute ischemic stroke (AIS) care in 7 European countries (France, Germany, Italy, the Netherlands, Spain, Sweden and the United Kingdom). The analysis compared the standard care for patients with AIS with alternative, improved care meeting the targets based on the SAP-E 2018-2030 (see “Targets Evaluated” section). The evaluation was conducted from the perspective of the national healthcare payer and adopted a lifetime time horizon to capture the long-term costs and health outcomes associated with stroke care. While this analysis evaluates the health economic impact of reaching these targets, it does not include an assessment of the requirements necessary for their achievement such as investments in infrastructure and delivery process modifications.

### Model structure

**Figure 1:**
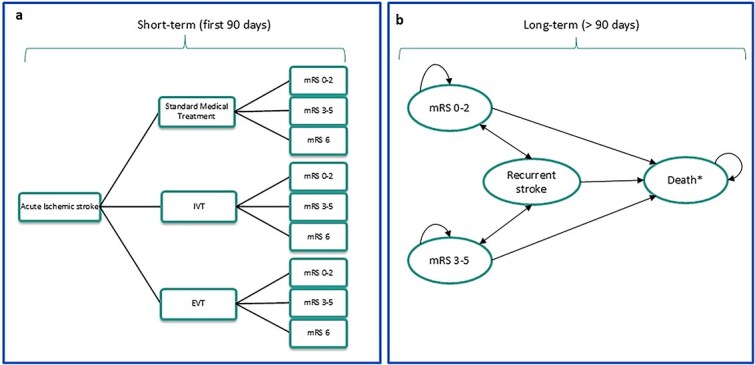
Model schematic. Overview of the health economic model, divided in the short-term model up to 90 days after the stroke (a) and the long-term modelling the life-time impact (>90 days) (b). Note: EVT = endovascular treatment; IVT = intravenous treatment; mRS = modified Rankin Scale Score. *Includes stroke-related mortality and general population mortality.

A schematic overview of the decision-analytic model is shown in Figure 1. A 2-stage model consisting of a decision tree (Figure 1a) and a Markov model (Figure 1b) was developed to simulate the progression of AIS patients in the first 90 days through to post-stroke long-term care, accounting for recurrent stroke, disability progression (ie, mRS 3–5) and mortality (ie, mRS 6). The model structure reflected the baseline stroke care pathway and alternative improved care pathways, as informed by clinical guidelines and published literature, and was validated by expert opinion. The decision-analytic model was constructed using Microsoft Excel.

A decision tree (Figure 1a) simulated 90-day functional outcomes, as measured by the modified Rankin Scale (mRS), for patients receiving one of 3 treatment strategies: standard medical treatment (including antiplatelet therapy, blood pressure management and treatment of underlying conditions), intravenous therapy (IVT) alone or endovascular therapy (EVT) (with or without IVT). The outcomes at 90 days were categorised into 3 groups: mRS 0–2 (functionally independent), mRS 3–5 (functionally dependent) and mRS 6 (death).

A Markov state-transition model (Figure 1b) was used to estimate long-term costs and QALYs over a lifetime horizon. The model comprised 4 health states defined by mRS categories (mRS 0–2, mRS3–5, mRS 6) and the occurrence of recurrent stroke (recurrent stroke). Transition probabilities of transitions between mRS 0–2, mRS3–5 and mRS 6 were derived from published literature and real-world data (see Table 1 for detailed data sources). Annual recurrent stroke risks varied by time since the initial event, that is, highest in the first year, and progressively lower in years 1–5, 6–10 and beyond 10 years.^11^ The death state accounted for both stroke-related and background population mortality sourced from national life tables.

**Table 1:** Model input parameters.

Parameters	Base-case value	Sensitivity analysis		References
		DSA range distribution	**Distribution + parameters (α,β)** ^**a**^ ^**,**^ ^**b**^	
*Short-term probabilities—baseline*				
Proportion of patients in mRS 0–2 after standard medical care	0.22	Beta^c^	220, 780	^8^
Proportion of patients in mRS 6 after standard medical care	0.23	Beta^c^	230, 770	^8^
Proportion of patients in mRS 0–2 after IVT	0.27	Beta^c^	270, 730	^8^
Proportion of patients in mRS 6 after IVT	0.19	Beta^c^	190, 810	^8^
Proportion of patients in mRS 0–2 after EVT	0.46	Beta^c^	460, 540	^8^
Proportion of patients in mRS 6 after EVT	0.14	Beta^c^	140, 860	^8^
*Short-term probabilities—intervention*				
Increase in the proportion of patients in MRS 0–2 per minute of reduction in time to treatment	0.0008^d^	NA	NA	^9^
Odds ratio of mortality (proportion in mRS 6 at 90 days), per 1-hour delay	1.16^e^	NA	95% CI, 1.05–1.28	^9^
*Annual transition probabilities—baseline (“From” state ➔ “To” state)*				
mRS 0–2 → mRS 0–2	0.992	NA	0.8928–1.0000	^10^
mRS 0–2 → mRS 3–5	0.000	NA	NA	^10^
mRS 0–2 → recurrent stroke—long term	0.000	NA	NA	^11^
mRS 0–2 → recurrent stroke year 1	0.111	NA	0.0999–0.1221	^11^
mRS 0–2 → recurrent stroke year 2–5	0.041	NA	0.0366–0.0448	^11^
mRS 0–2 → recurrent stroke year 6–10	0.027	NA	0.0243–0.0297	^11^
mRS 0–2 → death	0.008	NA	0.0072–0.0088	^10^
mRS 3–5 → mRS 0–2	0.000	NA	NA	^10^
mRS 3–5 → recurrent stroke—long term	0.000	NA	NA	^11^
mRS 3–5 → recurrent stroke—year 1	0.111	NA	0.0999–0.1221	^11^
mRS 3–5 → recurrent stroke—year 2–5	0.041	NA	0.0363–0.0448	^11^
mRS 3–5 → recurrent stroke—year 6–10	0.027	NA	0.0243–0.0297	^11^
mRS 3–5 → death	0.039	NA	0.0351–0.0429	^10^
Recurrent stroke → mRS 0–2	0.250	NA	0.2250–0.2750	^11^
Recurrent stroke → mRS 3–5	0.605	NA	0.5445–0.6655	^11^
Recurrent stroke → recurrent stroke	0.000	NA	NA	^11^
Recurrent stroke → death	0.145	NA	0.1305–0.1595	
Health-related quality of life (HRQoL)				
Utility mRS 0–2	0.71	Beta	0.70–0.72	^10^
Utility mRS 3–5	0.20	Beta	0.19–0.21	^10^
Utility mRS 6	0	NA	NA	^10^

^a^When a beta distribution was used for probabilistic analyses, it was assumed that α = the mean frequency out of 1000 samples and β = 1000 –α.

^b^When distribution is NA, the distribution parameters represent the ranges used for deterministic sensitivity analysis.

^c^Probabilities were sampled using constrained beta distributions to ensure that all probabilities remained positive and summed to one within each cycle.

^d^Calculated from the ARD of 4.8% per hour delay (4.8% per hour; 0.0008 (0.08%) per minute saved) for mRS proportion.^9^

^e^OR = 1 (no reduction in mortality) was assumed for the United Kingdom because OR for onset-to-groyne-time >270 minutes was not statistically significant.

Abbreviations: DSA = deterministic sensitivity analyses; EVT = endovascular treatment; IVT = intravenous treatment; mRS = modified Rankin Scale Score.

### Targets evaluated

The baseline level of stroke care was compared against alternative, improved stroke care meeting the targets specified in the SAP-E 2018-2030.^6^ Targets were selected based on the availability of reliable evidence linking their achievement to measurable improvements in health outcomes. Also, we prioritised targets that primarily affect a single set of model parameters to avoid assuming additive benefits across multiple care gaps.

Six improvement targets outlined below were evaluated, each addressing a different aspect of stroke care. Targets 1–4 focused on acute management and aligned directly with SAP-E goals: these were absolute targets that some countries may have already achieved depending on baseline performance. In contrast, Targets 5–6 reflected the broader SAP-E ambition to reduce the overall stroke burden by 10% by 2030. Separation into primary (Target 5) and secondary prevention (Target 6) was introduced for this analysis to enable a more granular assessment. These were relative targets that should be pursued across all countries regardless of baseline incidence levels.

1. **Increase treatment rates**

- Target 1: Raise the intravenous treatment (IVT) rate to 15%.
- Target 2: Raise the endovascular treatment (EVT) rate to 5%.

1. **Reduce onset-to-treatment times**

- Target 3: Reduce onset-to-needle time to <120 minutes.
- Target 4: Reduce onset-to-puncture (OTP) time to <200 minutes.

1. **Reduce stroke incidence**

- Target 5: Reduce the incidence of recurrent stroke by 10%, reflecting improvements in secondary prevention.
- Target 6: Reduce the incidence of first-time stroke by 10%, reflecting improved primary prevention.

For each of the countries included, we estimated the impact of achieving Targets 1–6 based on the SAP-E, compared with the baseline level of care. For each country, the impact of reaching each target was estimated by modifying the relevant parameter from the baseline levels while keeping all other parameters unchanged. For example, if the observed OTP time was 244 minutes, the comparative pathway simulated an OTP time of <200 minutes in line with the European target. Multiple clinical and economic outcomes were then evaluated for each of the targets met. If Targets 1–4 were already met, the outcomes were not reported. In the model, Target 5 was represented as a 10% reduction in the recurrent stroke rate among patients with prior AIS, while Target 6 simulated a 10% shift of the model population into the general population pathway—effectively reducing the overall incidence of first-time stroke while maintaining the total cohort size.

### Model input parameters

All input parameters for the model are listed in Table 1. Data sources included national stroke registries, peer-reviewed literature, publicly available healthcare databases and validation by clinical experts. All country-specific input parameters, like the incidence, onset-to-treatment times, costs and all-cause mortality are listed with specific references in Table S1.

The clinical efficacy, in terms of the distribution of mRS scores at 90 days, is based on data from Candio et al.^8^ The transitional probabilities and assumptions applied in the Markov model are based on Mohan et al. and Peultier et al.^10,11^ The model assumes that long-term transition probabilities remain constant over the patients’ lifetime after the first 90 days, except for a decreasing recurrent stroke rate and age-adjusted mortality. The probability of experiencing recurrent stroke was assumed to be the same regardless of mRS score. The annual probabilities of recurrent stroke in the first year, 1–5 years and 6–10 years for the Markov model were calculated based on the cumulative risk of stroke recurrence reported in Mohan et al.^11^ All-cause mortality was incorporated to reflect long-term survival from the WHO.^12^ It is also assumed that all patients in mRS 3–5 remain in mRS 3–5, without improving to mRS 0–2.

Direct medical costs associated with the first 90 days after stroke (decision tree model) and long-term care (>90 days) (Markov model) were based on the country-specific cost estimates for the first 90 days and long-term care reported in Moreu et al.,^13^ converted to 2023 euros (€) using healthcare-related inflation indices for each country. Currency conversion from USD to EUR was performed using the xe.com exchange rate (1 USD = 0.86 EUR) accessed on 21 August 2025.^14^ As Moreu et al. reported costs for each of the 6 mRS categories, weighted averages of the costs reported for relevant mRS categories were used for the short-term nodes and Markov states in this study. Long-term costs after 90 days were discounted using the country-specific rates recommended by the respective national healthcare payer. The costs for the specific treatment were included, but the costs for the intervention to change the care pathway were not. The input data, including references, are summarised in Table S1. Utility weights for all 7 countries were obtained from Peultier et al.^10^

### Outcomes

The primary outcome was the incremental cost-effectiveness ratio (ICER), representing the additional cost per quality-adjusted life-year (QALY) gained for the improved care pathway compared with baseline care. The ICER was based on the total lifetime costs of stroke care and QALYs. Secondary outcomes included functional outcomes as measured by the mRS such as the proportion of disabled (ie, mRS 3–5) and non-disabled (ie, mRS 0–2) patients, life-years and mortality.

All outcomes were reported separately for the short term (ie, first 90 days), long term (ie, lifetime after the first 90 days) and overall totals. Population-level outcomes were separately reported and estimated for an annual incident cohort at the national level by multiplying per-patient results by the number of addressable patients in each country, allowing comparison of the population-level impact of meeting targets across countries.

## Results

### Baseline performance of stroke care

Substantial variability was observed across the 7 countries in meeting the targets defined by the SAP-E 2018-2030. Baseline performance was defined using time-to-treatment data from 2015 to 2020 and treatment rate data from 2015 to 2016, based on the most recent comparable datasets available across countries (see Supplementary Appendix A). Based on these data, Germany was the only country meeting all assessed targets. The Netherlands and Sweden performed well on most indicators, yet both fell short of the recommended target for EVT rate. Spain met 2 of the predefined targets, while the United Kingdom and France each met one. Italy met none of the targets assessed in this study.

Although more recent data may indicate improvements in stroke care, these differences underscore significant disparities in access, treatment coverage and care delivery across Europe. A detailed overview of each country’s baseline performance is presented in Table 2. Two of the targets aims for a 10% reduction in stroke incidence across Europe; as this reflects a future prevention goal, it is not represented in the baseline performance table.

**Table 2:** Baseline national performance.

	Decrease onset-to-needle time	Decrease onset-to-puncture time	Increase IVT rate	Increase EVT rate
**Target** ^**a**^	**<120 minutes** ^**b**^	**<200 minutes** ^**b**^	**15%** ^**c**^	**5%** ^**c**^
**Germany**	**96**	**160**	**17.5**	**5.2**
**Spain**	**57**	**166**	**(7.5)**	**(3.6)**
**France**	**(153)**	**(244)**	**(9.2)**	**5.3**
**The Netherlands**	**85**	**180**	**20.6**	**(4.6)**
**United Kingdom**	**100**	**(378)**	**(11.7)**	**(0.5)**
**Italy**	**(160)**	**(225)**	**(7.4)**	**(1.7)**
**Sweden**	**84**	**127**	**15**	**(2.2)**

^a^Targets are based on “Action Plan for Stroke in Europe 2018-2030.”^6^

^b^Time to treatment values are based on 2015-2020 data.

^c^Treatment rates were based on 2015-2016 data. All input parameters with the matching references can be found in Supplementary Appendix.

Abbreviations: EVT = endovascular treatment; IVT = intravenous treatment; (xxx) target not met.

### Base-case results

The incremental costs and QALYs of meeting Targets 1–6 vs the baseline stroke care from the base-case analysis are presented in Table 3. A graphical comparison of the impact of meeting targets on QALYs and costs in comparison to the baseline care is also available in Supplementary Appendix Figures S1 and S2. The total and disaggregated costs and QALYs from deterministic analysis are shown in Supplementary Appendix.

**Table 3:** Incremental costs and QALYs over a lifetime of a patient per target.

	Germany	France	Italy	The Netherlands	Spain	United Kingdom	Sweden
*Target 1—Increase the intravenous treatment (IVT) rate to 15%*							
Incremental QALYs	NA	0.0152	0.0208	NA	0.0196	0.0074	NA
Incremental costs	NA	€36.12	€86.60	NA	€78.85	€62.95	NA
ICER^a^ (€/QALY)	NA	€2370.46	€4161.76	NA	€4019.00	€8493.43	NA
Change in % mRS 0–2^b^	NA	0.29	0.38	NA	0.37	0.16	NA
Change in % mRS 3–5	NA	−0.05	−0.07	NA	−0.08	−0.03	NA
*Target 2—Increase the endovascular treatment (EVT) rate to 5%*							
Incremental QALYs	NA	NA	0.0373	0.0058	0.0151	0.0418	0.0297
Incremental costs	NA	NA	€68.03	€26.71	−€114.49	€222.62	−€220.20
ICER^a^ (€/QALY)	NA	NA	€1822.60	€4606.93	Dominant	€5330.83	Dominant
Change in % mRS 0–2^b^	NA	NA	0.79	0.1	0.34	1.08	0.67
Change in % mRS 3–5	NA	NA	−0.49	−0.06	−0.21	−0.68	−0.42
*Target 3—Reduce onset-to-needle (OTN) time to <120 minutes*							
Incremental QALYs	NA	0.1314	0.1655	NA	NA	NA	NA
Incremental costs	NA	−€858.44	−€304.81	NA	NA	NA	NA
ICER^a^ (€/QALY)	NA	Dominant	Dominant	NA	NA	NA	NA
Change in % mRS 0–2^b^	NA	2.72	3.28	NA	NA	NA	NA
Change in % mRS 3–5	NA	−1.21	−1.44	NA	NA	NA	NA
*Target 4—Reduce onset-to-puncture (OTP) time to <200 minutes*							
Incremental QALYs	NA	0.1739	0.1050	NA	NA	0.4747	NA
Incremental costs	NA	−€1136.17	−€193.29	NA	NA	−€11,468.23	NA
ICER^a^ (€/QALY)	NA	Dominant	Dominant	NA	NA	Dominant	NA
Change in % mRS 0–2^b^	NA	3.6	2.08	NA	NA	14.32	NA
Change in % mRS 3–5	NA	−1.6	−0.91	NA	NA	−14.32	NA
*Target 5—Reduce the incidence of recurrent stroke by 10%*							
Incremental QALYs	0.0127	0.0108	0.0105	0.0162	0.0103	0.0082	0.0101
Incremental costs	−€53.43	€76.50	−€1.09	−€436.17	€308.17	−€483.69	−€265.62
ICER^a^ (€/QALY)	Dominant	€7103.83	Dominant	Dominant	€29,847.76	Dominant	Dominant
*Target 6—Reduce the incidence of first-time stroke by 10% (primary prevention)*							
Incremental QALYs	0.5334	0.4671	0.4877	0.6421	0.4589	0.3799	0.4495
Incremental costs	−€7449.16	−€7933.79	−€3402.54	−€6230.71	−€9788.16	−€7438.45	−€10,560.56
ICER^a^ (€/QALY)	Dominant	Dominant	Dominant	Dominant	Dominant	Dominant	Dominant

^a^ICER: Incremental cost-effectiveness ratio, that is, cost (€) per QALY gained. A “dominant” strategy generates greater QALYs whilst reducing costs.

^b^Absolute difference in percentage of patients in mRS 0–2.

Abbreviations: mRS = modified Rankin Score Scale; QALY = quality-adjusted life-years.

The results indicate that the most cost-effective strategies differ across countries, reflecting variation in existing stroke care, treatment uptake and cost structures. Achieving the proposed targets may either reduce or increase overall costs depending on the relative balance between long-term stroke care costs and first 90-day costs, including those associated with IVT and EVT, as well as cost differences linked to disability (mRS 3–5) and mortality (mRS 6) in respective countries. Reducing time to treatment is estimated to have the greatest impact in France, Italy and the United Kingdom, whereas increasing EVT rates to 5% yields the largest benefits in Sweden and Spain. In contrast, reducing recurrent stroke yields the largest gains in Germany and the Netherlands. These findings highlight country-specific unmet needs and opportunities to improve both health and economic outcomes.

#### Population-level outcomes

The model also estimates population-level costs and QALYs of meeting each target, accounting for country-specific population size and ischemic stroke incidence. An example of population-level incremental costs and QALYs of a 10% reduction in recurrent stroke (Target 5) across 7 countries is reported in Supplementary Appendix Figure S3. Population-level results were consistent with per-patient outcomes, as the latter were scaled to the incident population size whilst also incorporating general population mortality. Consequently, the greatest impact was observed in countries with the largest incident populations and for primary prevention of first-time stroke (Target 6), where a 10% reduction produced the most substantial health and economic benefits.

#### Deterministic sensitivity analysis

**Figure 2:**
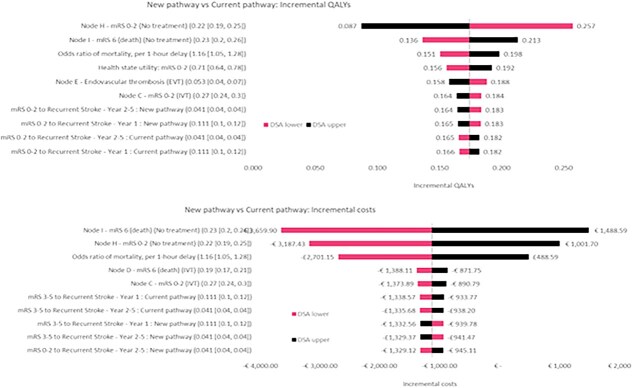
Tornado diagram—France: incremental QALYs and costs of meeting Target 4 (OTP <200 minutes). Target 4: reduce onset-to-puncture time to <200 minutes. Note: DSA = deterministic sensitivity analysis; EVT = endovascular treatment; IVT = intravenous treatment; mRS = modified Rankin Scale Score; OTP = onset-to-puncture time; QALY = quality-adjusted life-years.

Across all Targets 1–6, the deterministic sensitivity analysis shows that achieving targets remains dominant or cost-effective across the majority of ranges of parameter values. A few common parameters had the greatest impact on incremental costs and QALYs across targets, particularly the proportion of patients in mRS 0–2 and mRS 6 (death) among those who received standard medical treatments. This reflects the much larger share of patients treated with standard medical treatment compared with IVT or EVT. For time reduction targets (Targets 3–4), the odds ratio of 90-day mortality per 1-hour treatment delay was among the most influential parameters, as reductions in early mortality can significantly alter long-term health and cost outcomes (Figure 2). In addition, the results from probabilistic sensitivity analyses are available in Figures S4 and S5  in  Supplementary  Appendix.

## Discussion

This cost-effectiveness analysis assessed the health and economic outcomes of enhancing stroke care across multiple European countries to identify which improvement targets would yield the greatest clinical and economic benefits. This study shows that the most cost-effective strategies to improve stroke care differ by country, depending on the baseline level of stroke care and costs. Reducing the incidence of stroke (Targets 5 and 6) consistently yields the largest overall benefits in terms of both costs and health outcomes in all countries. This finding shows that investing in stroke prevention will not only reduce the future burden of acute care but also alleviate the long-term costs associated with disability and dependency.

Substantial variability was observed in stroke care delivery across Europe, suggesting that tailored national strategies are needed to achieve major benefits for patients, healthcare systems and society.^4,6^ Germany was the only country meeting all benchmarks, while other showed mixed or limited achievement, with Italy not meeting any. Our findings underscore the differences in access to care and the urgent need for targeted interventions to close these gaps and optimise outcomes, as in line with earlier publications.^1,4–6^ Our model shows country-specific data regarding the unmet needs in stroke care, consequences of the unmet needs, opportunities to improve and impact on the clinical and health economic outcomes.

Our study shows that the most cost-effective strategies to improve stroke care differ by country, depending on the baseline level of stroke care and costs. Reducing the incidence of stroke consistently yields the largest overall benefits in terms of both costs and health outcomes in all countries. This finding reinforces the importance of primary prevention strategies, such as controlling cardiovascular risk factors, promoting healthy lifestyles and early detection and treatment of atrial fibrillation and hypertension.^15–17^ Investing in prevention will not only reduce the future burden of acute care but also alleviate long-term costs associated with disability and dependency.

At the same time, the model highlights important differences in which stroke care interventions create the greatest additional value. In France, Italy and the United Kingdom, shortening the time to treatment is the most impactful strategy. This aligns with clinical evidence that “time is brain”^18^ and that faster treatment translates directly into improved clinical outcomes and lower long-term costs.^18–20^ Investments in pre-hospital triage, increasing the number of stroke units and streamlined in-hospital processes could therefore deliver high returns in these countries.

In contrast, in Sweden and Spain, increasing endovascular treatment rates represents the most cost-effective pathway. Although these countries already provide strong acute care, expanding access to EVT could unlock substantial additional health gains and reduce the healthcare costs. Policies that support the training and retention of neurointerventional specialists, increase the number of stroke units and strengthen referral networks will be critical to realise this potential.

Finally, in Germany and the Netherlands, reducing recurrent stroke emerged as the most impactful strategy. This finding underscores the important role of secondary prevention (eg, improved hypertension, promoting healthy lifestyles and atrial fibrillation detection and management^15–17^) and the need to optimise adherence to medical therapies, ensure follow-up and integrate lifestyle interventions. Given the high costs and disability burden associated with recurrent events, interventions in this area will have a high impact.

Similar stroke strategies have been developed globally. Australia and the United States have emphasised the importance of system-level performance targets and economic evaluation to improve stroke care.^21–23^ These examples reinforce the need for robust modelling frameworks that link clinical targets to economic consequences, supporting informed policy and investment decisions.

This study also has several limitations. First, all input parameters were derived from published literature, so the analysis depended on the accuracy and availability of these data, which vary across countries. In some cases, simplifying model assumptions also had to be made because of data gaps (eg, grouping mRS categories, risk of recurrent stroke). In addition, baseline stroke care performance was based on the latest multi-country data available at the time of review, some of which date back to 2015. As a result, these estimates may not fully capture more recent improvements in stroke care in some countries. Second, this study examined only a subset of targets from the SAP-E 2018-2030. Whilst stroke units remain a cornerstone of high-quality stroke care with wide-reaching impact across multiple targets, the target of expanding stroke unit capacity was not evaluated in this study. This exclusion aimed to minimise overlap between targets, as stroke units can simultaneously influence treatment rates, time to treatment and thus patient outcomes. We recommend that future research assess strategies that impact multiple targets, such as increasing the number of stroke units, to fully capture their comprehensive contribution to improved outcomes.^24^ Third, some model assumptions and calculations were required to enable a consistent multi-country analysis and may not fully capture local heterogeneity. These assumptions were informed by published evidence available, including the presumed linear relationship between treatment delays and clinical outcomes. In addition, to facilitate efficient probabilistic sensitivity analysis across multiple countries, multinomial probabilities were parameterised using independent beta distributions with constraints to ensure sampled values were non-negative and summed to one, rather than using a Dirichlet distribution. While this represents a pragmatic modelling choice, it may simplify the joint uncertainty structure of probabilities. Fourth, while the health economic model incorporates both short-term and long-term outcomes through a decision tree and Markov framework, it is still a simplification of real-world care pathways. For example, the model assumes that transitions from functional independence to dependency occur only through recurrent stroke, although, in reality, dependency may arise from a broader range of clinical conditions. This assumption was necessary due to limited availability of consistent data across countries, but it does not capture other clinical causes of dependency and should be considered when interpreting the results. Finally, as outlined in the “Methods” section, this study focuses exclusively on estimating the potential impact of achieving the defined targets, without addressing the specific interventions required to reach them. The associated implementation strategies and costs were not included, as these depend on national, regional and organisational factors that vary widely across healthcare systems. Other important societal aspects of stroke care such as indirect, societal costs and social support were not fully incorporated, yet likely to contribute substantially to long-term outcomes and costs.

For policymakers, our analysis provides clear evidence that investing in stroke care delivers substantial clinical and economic benefits. While reducing stroke incidence should remain the overarching priority across all health systems, the most cost-effective strategies to close gaps vary between countries. By implementing strategies to reduce the time to treatment, increase treatment rates and enhance prevention, countries can not only meet SAP-E targets but also reduce the societal and economic burden of stroke. Policymakers should adopt a tailored approach, focusing investments on the interventions that promise the highest return in their specific context. At the European level, collaboration and knowledge-sharing will be key to accelerating progress, while health economic evidence can guide resource allocation towards interventions that combine clinical effectiveness with sustainability. Future research should explore the feasibility, resource implications and potential innovations required to achieve these targets in different healthcare systems.

## Conclusion

In conclusion, our findings demonstrate that reaching SAP-E 2030 targets has the potential to deliver major health and economic gains across Europe. The optimal intervention in stroke care differs by country, reflecting existing levels of care and system organisation. By aligning investments with these priorities, countries can move closer to equitable, efficient and high-quality stroke care for all.

## Supplementary Material

aakag041_Supplementary_material
