# Supplementary material for: Enhancing stroke care in Europe: cost-effectiveness analysis of strategies addressing gaps in acute ischemic stroke care
Source: Eur Stroke J. 2026 May 19;11(5):aakag041. doi: 10.1093/esj/aakag041 (PMC13184963; doi:10.1093/esj/aakag041)
Supplement: aakag041_Supplementary_material [file aakag041_supplementary_material.zip › Suppl_R2_clean_ESO_25_1093_Manuscript Enhancing Stroke Care in Europe_v3.01.docx]

**Supplementary Appendices**

For the manuscript:

Enhancing Stroke Care in Europe: Cost-Effectiveness Analysis of Strategies Addressing Gaps in Acute Ischemic Stroke Care

Bianca de Greef,^1^ Ji-Hee Youn,^1^ Henri vanden Bavière,^1^ Marjan Hummel,^1^ Angelique Balguid,^2^ Francesca Pezzella,^3^ Hanne Christensen,^4^ and Wim van Zwam.^5^

^1^ Chief Medical Office – Health Economics and Outcomes Research, Philips, Amsterdam, the Netherlands

^2^ Image-Guided Therapy Systems, Philips, Best, the Netherlands

^3^ Stroke Unit, Department of Neuroscience, S Camillo Forlanini Hospital, Rome, Italy

^4^ Department of Neurology, Copenhagen University Hospital, Bispebjerg, Denmark

^5^ Department of Radiology and Nuclear Medicine, Maastricht University Medical Center+, the Netherlands.

1. **Model inputs**

***Tabel S1 – Country Specific Input data***

**France**

| **Parameters** | **Base-case value** | **Probabilistic value** | | **Reference** |
| --- | --- | --- | --- | --- |
|  |  | **Distribution** | **Distribution parameters** |  |
| Clinical data | | | | |
| Baseline Incidence AIS (rate per 100,000 inhabitants) | 105.78 | NA | NA | (1) |
| Average onset-to-door time, min | 86 | NA | NA | (2) |
| Average door-to-needle time, min | 67 | NA | NA | (2) |
| Average door-to-puncture time, min | 158 | NA | NA | (2,3) |
| Average onset-to-needle time, min | 153 | NA | NA | (2) |
| Average onset-to-puncture time, min | 244 | NA | NA | (3) |
| Proportion of patients receiving IVT | 0.092 | Beta | 0.075-0.111 | (4) |
| Proportion of patients receiving EVT | 0.053 | Beta | 0.040-0.068 | (4) |
| Cost data |  |  |  |  |
| Costs mRS 0-2 after standard medical treatment | €6,510 | Gamma | €5,296-€7,845 | (5) |
| Costs mRS 3-5 after standard medical treatment | €9,637 | Gamma | €7,841-€11,616 | (5) |
| Costs mRS 6 after standard medical treatment | €11,389 | Gamma | €9,267-€13,728 | (5) |
| Costs mRS 0-2 after IVT | €7,282 | Gamma | €5,925-€8,777 | (5) |
| Costs mRS 3-5 after IVT | €10,404 | Gamma | €8,465-€12,540 | (5) |
| Costs mRS 6 after IVT | €12,162 | Gamma | €9,895-€14,658 | (5) |
| Costs mRS 0-2 after EVT | €15,910 | Gamma | €12,945-€19,176 | (5) |
| Costs mRS 3-5 after EVT | €19,015 | Gamma | €15,471-€22,918 | (5) |
| Costs mRS 6 after EVT | €20,790 | Gamma | €16,915-€25,057 | (5) |
| Long term care costs mRS 0-2 | €781 | Gamma | €669-€902 | (5) |
| Long term care costs mRS 3-5 | €9,632 | Gamma | €15,989-€17,288 | (5) |
| Costs recurrent stroke | €13,583 | Gamma | €13,036-€14,141 | (5) |
| All-cause mortality | | | | |
| Age 70-75 | 0.0153 | NA | NA | (6) |
| Age 75-80 | 0.0231 | NA | NA | (6) |
| Age 80-85 | 0.0425 | NA | NA | (6) |
| Age 85+ | 0.2644 | NA | NA | (6) |
| Other | | | | |
| Discount effect | 4% | NA | NA | (7) |
| Discount costs | 4% | NA | NA | (7) |
| Average age stroke | 74.2 | NA | NA | (8) |
| Inflation index from 2015 to 2023 | 1.0189 | NA | NA | (9) |

*AIS; Acute Ischemic Stroke, EVT; endovascular treatment, IVT; intravenous treatment, mRS; modified Rankin Scale Score, QALY; quality adjusted life years.*

**Germany**

| Parameters | Base-case value | Probabilistic value | | Reference |
| --- | --- | --- | --- | --- |
|  |  | **Distribution** | **Distribution parameters** |  |
| Clinical data | | | | |
| Baseline Incidence AIS (rate per 100,000 inhabitants) | 177.49 | NA | NA | (1) |
| Average onset-to-door time, min | 70.2 | NA | NA | (10) |
| Average door-to-needle time, min | 30 | NA | NA | (10) |
| Average door-to-puncture time, min | 79.8 | NA | NA | (10) |
| Average onset-to-needle time, min | 96 | NA | NA | (10) |
| Average onset-to-puncture time, min | 160.2 | NA | NA | (10) |
| Proportion of patients receiving IVT | 0.175 | Beta | 0.142-0.188 | (4) |
| Proportion of patients receiving EVT | 0.052 | Beta | 0.051-0.081 | (4) |
| Cost data |  |  |  |  |
| Costs mRS 0-2 after standard medical treatment | €6,388 | Gamma | €5,198-€7,700 | (5) |
| Costs mRS 3-5 after standard medical treatment | €20,101 | Gamma | €16,355-€24,227 | () |
| Costs mRS 6 after standard medical treatment | €1,708 | Gamma | €1,389-€2,058 | (5) |
| Costs mRS 0-2 after IVT | €10,539 | Gamma | €8,575-€12,703 | (5) |
| Costs mRS 3-5 after IVT | €20,534 | Gamma | €16,707-€24,749 | (5) |
| Costs mRS 6 after IVT | €1,708 | Gamma | €1,389-€2,058 | (5) |
| Costs mRS 0-2 after EVT | €14,233 | Gamma | €11,580-€17,155 | (5) |
| Costs mRS 3-5 after EVT | €23,753 | Gamma | €19,326-€28,629 | (5) |
| Costs mRS 6 after EVT | €5,742 | Gamma | €4,672-€6,921 | (5) |
| Long term care costs mRS 0-2 | €1,752 | Gamma | €1,637-€1,870 | (5) |
| Long term care costs mRS 3-5 | €12,121 | Gamma | €11,480-€12,779 | (5) |
| Costs recurrent stroke | €16,152 | Gamma | €15,605-€16,709 | (5) |
| All-cause mortality | | | | |
| Age 70-75 | 0.0209 | NA | NA | (6) |
| Age 75-80 | 0.0312 | NA | NA | (6) |
| Age 80-85 | 0.0567 | NA | NA | (6) |
| Age 85+ | 0.2644 | NA | NA | (6) |
| Other | | | | |
| Discount effect | 3% | NA | NA | (11) |
| Discount costs | 3% | NA | NA | (11) |
| Average age stroke | 72 | NA | NA | (12) |
| Inflation index from 2021 to 2023 | 1.0438 | NA | NA | (13) |

*AIS; Acute Ischemic Stroke, EVT; endovascular treatment, IVT; intravenous treatment, mRS; modified Rankin Scale Score, QALY; quality adjusted life years.*

**Italy**

| Parameters | Base-case value | Probabilistic value | | Reference |
| --- | --- | --- | --- | --- |
|  |  | **Distribution** | **Distribution parameters** |  |
| Clinical data | | | | |
| Baseline Incidence AIS (rate per 100,000 inhabitants) | 113.26 | NA | NA | (1) |
| Average onset-to-door time, min | 75 | NA | NA | (14) |
| Average door-to-needle time, min | 75 | NA | NA | (14) |
| Average door-to-puncture time, min | 128 | NA | NA | (14) |
| Average onset-to-needle time, min | 160 | NA | NA | (14) |
| Average onset-to-puncture time, min | 225 | NA | NA | (14) |
| Proportion of patients receiving IVT | 0.074 | Beta | 0.059-0.091 | (4) |
| Proportion of patients receiving EVT | 0.017 | Beta | 0.010-0.026 | (4) |
| Cost data |  |  |  |  |
| Costs mRS 0-2 after standard medical treatment | €3,237 | Gamma | €2,634-€3,901 | (5) |
| Costs mRS 3-5 after standard medical treatment | €4,343 | Gamma | €3,534-€5,235 | (5) |
| Costs mRS 6 after standard medical treatment | €4,777 | Gamma | €3,887-€5,758 | (5) |
| Costs mRS 0-2 after IVT | €4,342 | Gamma | €3,533-€5,234 | (5) |
| Costs mRS 3-5 after IVT | €5,264 | Gamma | €4,283-€6,344 | (5) |
| Costs mRS 6 after IVT | €5,716 | Gamma | €4,651-€6,889 | (5) |
| Costs mRS 0-2 after EVT | €9,409 | Gamma | €7,656-€11,341 | (5) |
| Costs mRS 3-5 after EVT | €10,274 | Gamma | €8,359-€12,383 | (5) |
| Costs mRS 6 after EVT | €10,783 | Gamma | €8,774-€12,997 | (5) |
| Long term care costs mRS 0-2 | €587 | Gamma | €476-€709 | (5) |
| Long term care costs mRS 3-5 | €6,163 | Gamma | €5,530-€6,829 | (5) |
| Costs recurrent stroke | €7,054 | Gamma | €6,512-€7,616 | (5) |
| All-cause mortality | | | | |
| Age 70-75 | 0.0159 | NA | NA | (6) |
| Age 75-80 | 0.0257 | NA | NA | (6) |
| Age 80-85 | 0.0501 | NA | NA | (6) |
| Age 85+ | 0.2644 | NA | NA | (6) |
| Other | | | | |
| Discount effect | 3% | NA | NA | (15) |
| Discount costs | 3% | NA | NA | (15) |
| Average age stroke | 74.6 | NA | NA | (16) |
| Inflation index from 2015 to 2023 | 1.0700 | NA | NA | (17) |

*AIS; Acute Ischemic Stroke, EVT; endovascular treatment, IVT; intravenous treatment, mRS; modified Rankin Scale Score, QALY; quality adjusted life years.*

**The Netherlands**

| Parameters | Base-case value | Probabilistic value | | Reference |
| --- | --- | --- | --- | --- |
|  |  | **Distribution** | **Distribution parameters** |  |
| Clinical data | | | | |
| Baseline Incidence AIS (rate per 100,000 inhabitants) | 112.7 | NA | NA | (1) |
| Average onset-to-door time, min | 57 | NA | NA | (18) |
| Average door-to-needle time, min | 28 | NA | NA | (18) |
| Average door-to-puncture time, min | 123 | NA | NA | (18) |
| Average onset-to-needle time, min | 85 | NA | NA | (18) |
| Average onset-to-puncture time, min | 180 | NA | NA | (18) |
| Proportion of patients receiving IVT | 0.206 | Beta | 0.182-0.232 | (4) |
| Proportion of patients receiving EVT | 0.046 | Beta | 0.034-0.060 | (4) |
| Cost data |  |  |  |  |
| Costs mRS 0-2 after standard medical treatment | €8,463 | Gamma | €6,886-€10,200 | (5) |
| Costs mRS 3-5 after standard medical treatment | €21,408 | Gamma | €17,418-€25,803 | (5) |
| Costs mRS 6 after standard medical treatment | €17,775 | Gamma | €14,463-€21,424 | (5) |
| Costs mRS 0-2 after IVT | €8,607 | Gamma | €7,003-€10,374 | (5) |
| Costs mRS 3-5 after IVT | €19,574 | Gamma | €15,926-€23,592 | (5) |
| Costs mRS 6 after IVT | €18,358 | Gamma | €14,937-€22,126 | (5) |
| Costs mRS 0-2 after EVT | €20,137 | Gamma | €16,384-€24,471 | (5) |
| Costs mRS 3-5 after EVT | €30,026 | Gamma | €24,430-€36,189 | (5) |
| Costs mRS 6 after EVT | €30,101 | Gamma | €24,491-€36,281 | (5) |
| Long term care costs mRS 0-2 | €751 | Gamma | €639-€767 | (5) |
| Long term care costs mRS 3-5 | €5,310 | Gamma | €4,680-€5,979 | (5) |
| Costs recurrent stroke | €21,827 | Gamma | €21,278-€22,383 | (5) |
| All-cause mortality | | | | |
| Age 70-75 | 0.0183 | NA | NA | (6) |
| Age 75-80 | 0.0302 | NA | NA | (6) |
| Age 80-85 | 0.0562 | NA | NA | (6) |
| Age 85+ | 0.2644 | NA | NA | (6) |
| Other | | | | |
| Discount effect | 1.5% | NA | NA | (19) |
| Discount costs | 3% | NA | NA | (19) |
| Average age stroke | 71 | NA | NA | (20) |
| Inflation index from 2000 to 2023 | 1.2892 | NA | NA | (21) |

*AIS; Acute Ischemic Stroke, EVT; endovascular treatment, IVT; intravenous treatment, mRS; modified Rankin Scale Score, QALY; quality adjusted life years.*

**Spain**

| Parameters | Base-case value | Probabilistic value | | Reference |
| --- | --- | --- | --- | --- |
|  |  | **Distribution** | **Distribution parameters** |  |
| Clinical data | | | | |
| Baseline Incidence AIS (rate per 100,000 inhabitants) | 117.09 | NA | NA | (1) |
| Average onset-to-door time, min | 89 | NA | NA | (22) |
| Average door-to-needle time, min | 163.5 | NA | NA | (23) |
| Average door-to-puncture time, min | 77 | NA | NA | (22) |
| Average onset-to-needle time, min | 56.5 | NA | NA | (23) |
| Average onset-to-puncture time, min | 166 | NA | NA | (22) |
| Proportion of patients receiving IVT | 0.075 | Beta | 0.060-0.092 | (4) |
| Proportion of patients receiving EVT | 0.036 | Beta | 0.025-0.048 | (4) |
| Cost data |  |  |  |  |
| Costs for standard medical treatment | €536 | Gamma | €436-€647 | (24) |
| Costs for IVT | €1,421 | Gamma | €1,156-€1,713 | (24) |
| Costs for EVT | €7,457 | Gamma | €6,067-€8,987 | (24) |
| Acute care costs mRS 0-2 | €4,444 | Gamma | €3,616-€5,357 | (24) |
| Acute care costs mRS 3-5 | €5,810 | Gamma | €4,728-€7,003 | (24) |
| Acute care costs mRS 6 | €3,361 | Gamma | €2,735-€4,051 | (24) |
| Long term care costs mRS 0-2 | €1,272 | Gamma | €1,158-€1,391 | (24) |
| Long term care costs mRS 3-5 | €21,338 | Gamma | €20,693-€21,992 | (24) |
| Costs recurrent stroke | €8,950 | Gamma | €8,406-€9,511 | (24) |
| All-cause mortality | | | | |
| Age 70-75 | 0.0145 | NA | NA | (6) |
| Age 75-80 | 0.0254 | NA | NA | (6) |
| Age 80-85 | 0.047 | NA | NA | (6) |
| Age 85+ | 0.2644 | NA | NA | (6) |
| Other | | | | |
| Discount effect | 3% | NA | NA | (24) |
| Discount costs | 3% | NA | NA | (24) |
| Average age stroke | 75 | NA | NA | (25) |
| Inflation index from 2016 to 2023 | 1.0646 | NA | NA | (26) |

*AIS; Acute Ischemic Stroke, EVT; endovascular treatment, IVT; intravenous treatment, mRS; modified Rankin Scale Score, QALY; quality adjusted life years.*

**Sweden**

| Parameters | Base-case value | Probabilistic value | | Reference |
| --- | --- | --- | --- | --- |
|  |  | **Distribution** | **Distribution parameters** |  |
| Clinical data | | | | |
| Baseline Incidence AIS (rate per 100,000 inhabitants) | 137.12 | NA | NA | (1) |
| Average onset-to-door time, min | 86 | NA | NA | (27) |
| Average door-to-needle time, min | 13 | NA | NA | (27) |
| Average door-to-puncture time, min | 62 | NA | NA | (27) |
| Average onset-to-needle time, min | 84 | NA | NA | (27) |
| Average onset-to-puncture time, min | 127 | NA | NA | (27) |
| Proportion of patients receiving IVT | 0.15 | Beta | 0.129-0.173 | (4) |
| Proportion of patients receiving EVT | 0.022 | Beta | 0.014-0.032 | (4) |
| Cost data |  |  |  |  |
| Costs mRS 0-2 after standard medical treatment | €11,286 | Gamma | €9,183-€13,603 | (5) |
| Costs mRS 3-5 after standard medical treatment | €32,828 | Gamma | €26,710-€39,567 | (5) |
| Costs mRS 6 after standard medical treatment | €21,501 | Gamma | €17,494-€25,915 | (5) |
| Costs mRS 0-2 after IVT | €12,211 | Gamma | €9,935-€14,718 | (5) |
| Costs mRS 3-5 after IVT | €32,130 | Gamma | €26,142-€38,725 | (5) |
| Costs mRS 6 after IVT | €22,426 | Gamma | €18,247-€27,030 | (5) |
| Costs mRS 0-2 after EVT | €19,621 | Gamma | €15,964-€23,649 | (5) |
| Costs mRS 3-5 after EVT | €38,113 | Gamma | €31,010-€45,937 | (5) |
| Costs mRS 6 after EVT | €29,836 | Gamma | €24,476-€35,961 | (5) |
| Long term care costs mRS 0-2 | €1,090 | Gamma | €977-€1,210 | (5) |
| Long term care costs mRS 3-5 | €16,322 | Gamma | €15,679-€16,978 | (5) |
| Costs recurrent stroke | €28,889 | Gamma | €28,339-€29,444 | (5) |
| All-cause mortality | | | | |
| Age 70-75 | 0.0165 | NA | NA | (6) |
| Age 75-80 | 0.0284 | NA | NA | (6) |
| Age 80-85 | 0.0529 | NA | NA | (6) |
| Age 85+ | 0.2644 | NA | NA | (6) |
| Other | | | | |
| Discount effect | 3% | NA | NA | (28) |
| Discount costs | 3% | NA | NA | (28) |
| Average age stroke | 75.5 | NA | NA | (29) |
| Inflation from 2015 to 2023 | 1.1098 | NA | NA | (30) |

*AIS; Acute Ischemic Stroke, EVT; endovascular treatment, IVT; intravenous treatment, mRS; modified Rankin Scale Score, QALY; quality adjusted life years.*

**United Kingdom**

| Parameters | Base-case value | Probabilistic value | | Reference |
| --- | --- | --- | --- | --- |
|  |  | **Distribution** | **Distribution parameters** |  |
| Clinical data | | | | |
| Baseline Incidence AIS (rate per 100,000 inhabitants) | 106.59 | NA | NA | (1) |
| Average onset-to-door time, min | 70 | NA | NA | (31) |
| Average door-to-needle time, min | 33 | NA | NA | (31) |
| Average door-to-puncture time, min | 308 | NA | NA | (32) |
| Average onset-to-needle time, min | 100 | NA | NA | (31) |
| Average onset-to-puncture time, min | 378 | NA | NA | (32) |
| Proportion of patients receiving IVT | 0.117 | Beta | 0.098-0.138 | (4) |
| Proportion of patients receiving EVT | 0.005 | Beta | 0.002-0.010 | (4) |
| Cost data |  |  |  |  |
| Costs mRS 0-2 after standard medical treatment | €6,812 | Gamma | €5,542-€8,210 | (5) |
| Costs mRS 3-5 after standard medical treatment | €38,945 | Gamma | €31,687-€46,940 | (5) |
| Costs mRS 6 after standard medical treatment | €5,781 | Gamma | €4,703-€6,967 | (5) |
| Costs mRS 0-2 after IVT | €9,016 | Gamma | €7,336-€10,867 | (5) |
| Costs mRS 3-5 after IVT | €39,377 | Gamma | €32,039-€47,461 | (5) |
| Costs mRS 6 after IVT | €8,060 | Gamma | €6,558-€9,714 | (5) |
| Costs mRS 0-2 after EVT | €20,015 | Gamma | €16,285-€24,124 | (5) |
| Costs mRS 3-5 after EVT | €48,830 | Gamma | €39,730-€58,854 | (5) |
| Costs mRS 6 after EVT | €19,172 | Gamma | €15,599-€23,107 | (5) |
| Long term care costs mRS 0-2 | €1,516 | Gamma | €1,401-€1,634 | (5) |
| Long term care costs mRS 3-5 | €8,596 | Gamma | €7,959-€9,258 | (5) |
| Costs recurrent stroke | €29,689 | Gamma | €29,140-€30,244 | (5) |
| All-cause mortality | | | | |
| Age 70-75 | 0.0187 | NA | NA | (6) |
| Age 75-80 | 0.0308 | NA | NA | (6) |
| Age 80-85 | 0.0549 | NA | NA | (6) |
| Age 85+ | 0.2644 | NA | NA | (6) |
| Other | | | | |
| Discount effect | 3.5% | NA | NA | (33) |
| Discount costs | 3.5% | NA | NA | (33) |
| Average age stroke | 77 | NA | NA | (34) |
| Inflation index from 2015 to 2023 | 1.2128 | NA | NA | (35) |

*AIS; Acute Ischemic Stroke, EVT; endovascular treatment, IVT; intravenous treatment, mRS; modified Rankin Scale Score, QALY; quality adjusted life years.*

1. **Model Results**

***Figure S1 – Incremental QALYs and incremental costs of meeting targets 1-6***

**Incremental QALYs and incremental costs of meeting Targets 1-6**

| 1. **Incremental QALYs of meeting Targets 1-6** |
| --- |
| 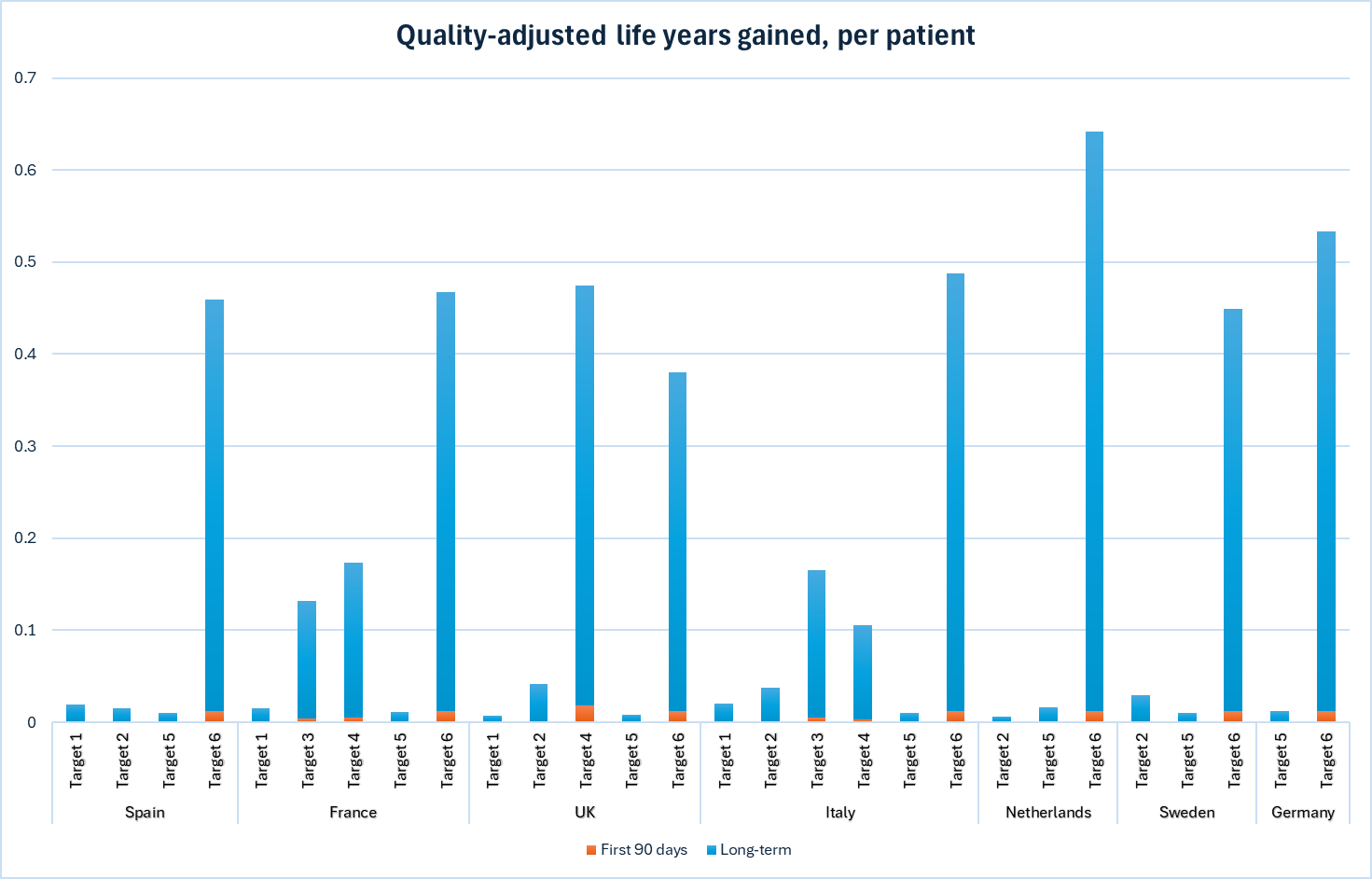 |
| 1. **Incremental costs of meeting Targets 1-6** |
| 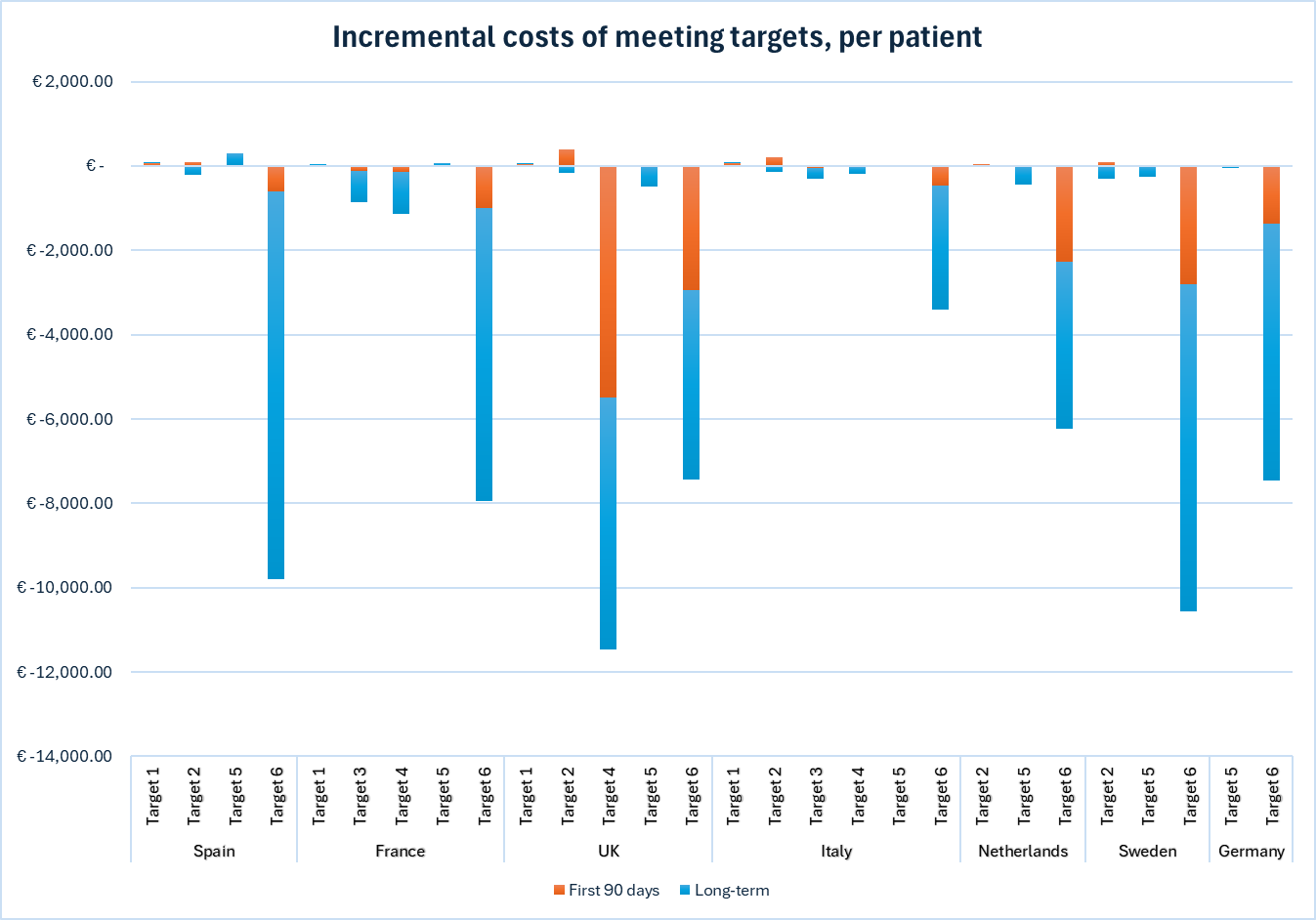 |

*Target 1: increase intravenous treatment rate to 15%, Target 2: increase endovascular treatment rate to 5%, Target 3: Reduce onset-to-needle time to <120 minutes, Target 4: reduce onset-to-puncture time to <200 minutes, Target 5: reduce incidence of recurrent stroke by 10%, and Target 6: reduce incidence of first-time stroke by 10%.*

***Figure S2 – Incremental QALYs and incremental costs of meeting targets 1-6***

| 1. **Incremental QALYs of meeting Targets 1-6** |
| --- |
| 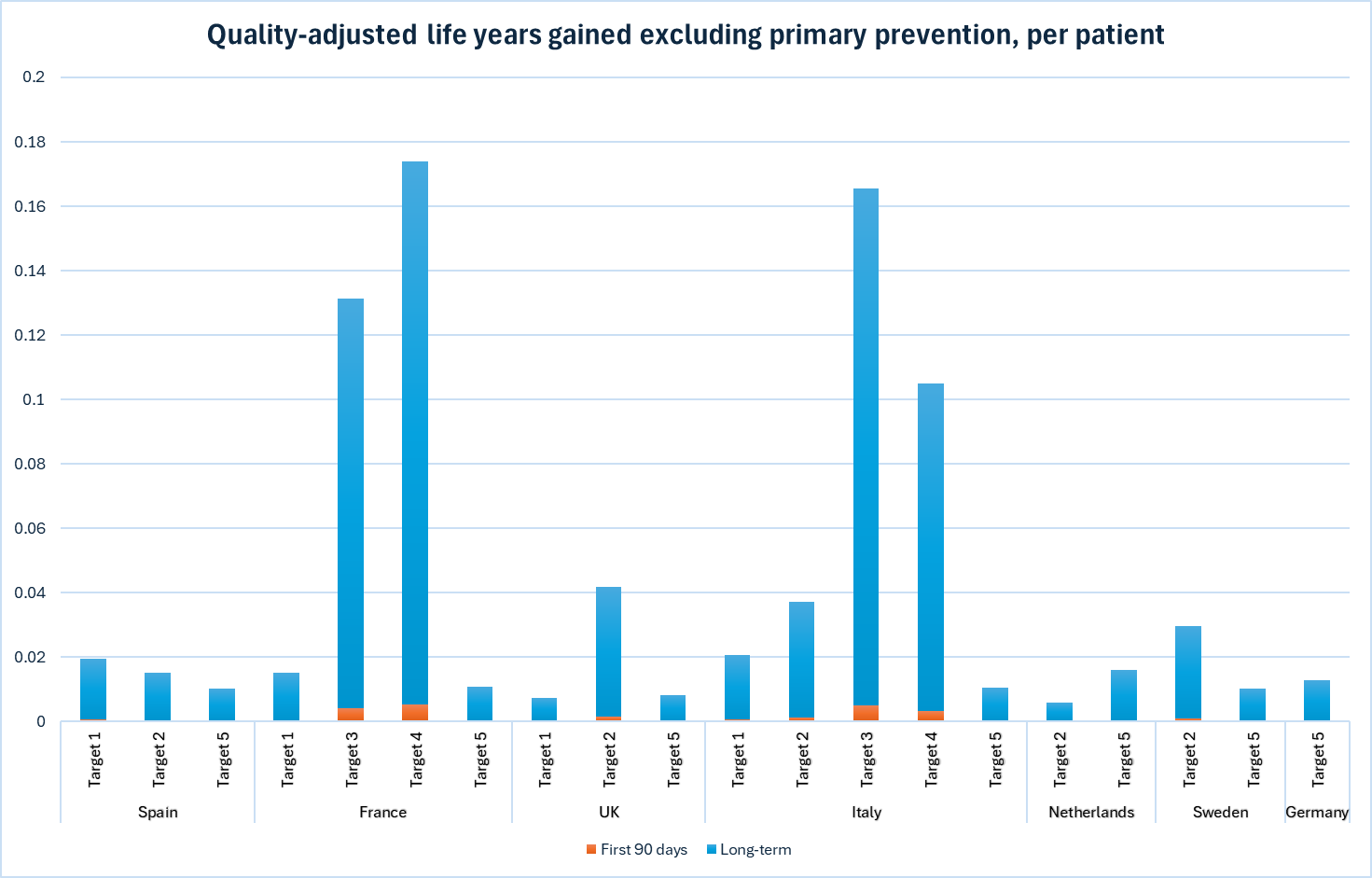 |
| 1. **Incremental costs of meeting Targets 1-6*** |
| 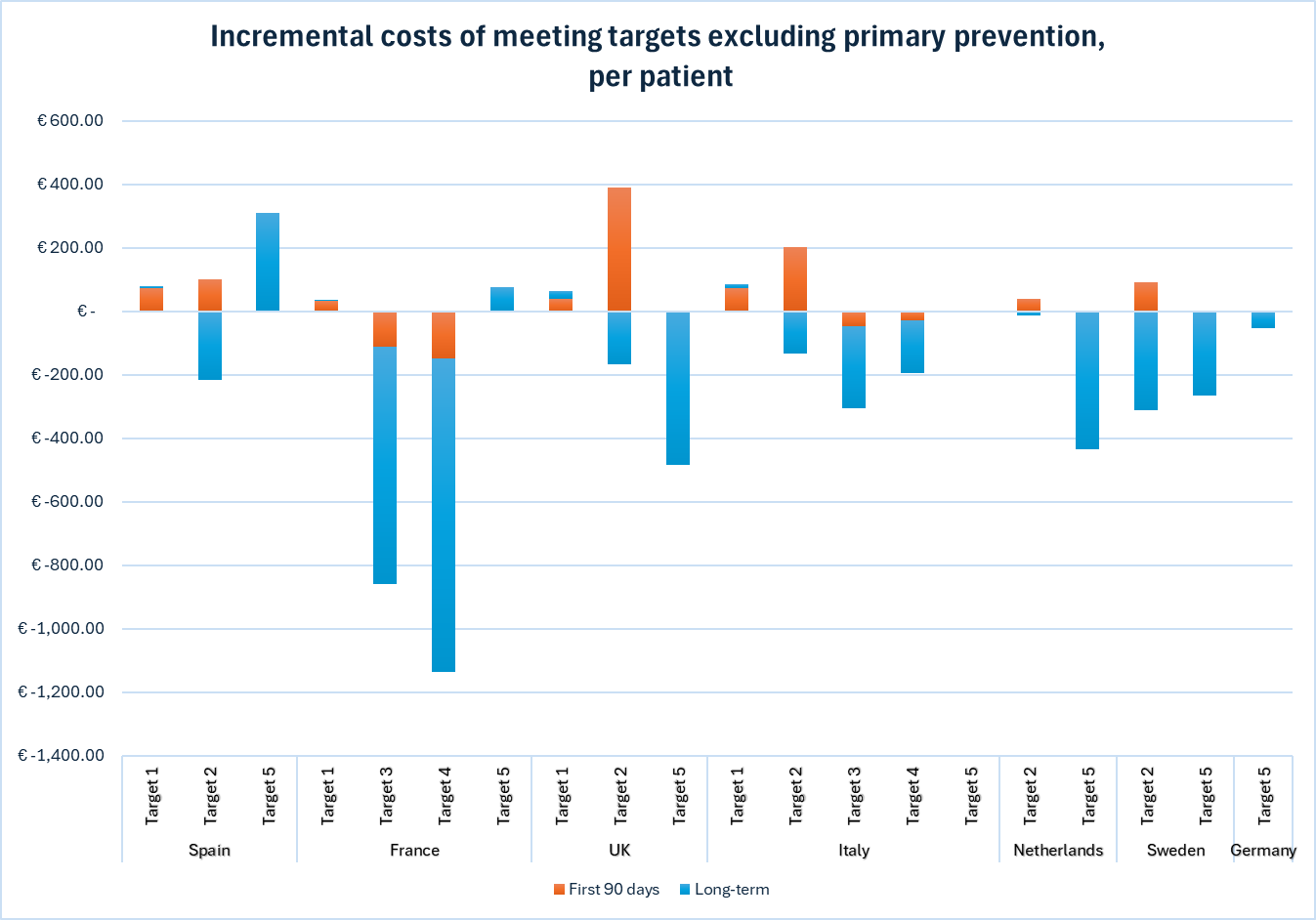 |

*** *Achieving the proposed targets may results in either positive or negative incremental costs, depending on the relative balance between long-term stroke care costs and first 90-day costs, including those associated with IVT and EVT, as well as cost differences linked to disability (mRS 3-5) and mortality (mRS 6) in respective countries; Target 1: increase intravenous treatment rate to 15%, target 2: increase endovascular treatment rate to 5%, target 3: Reduce onset-to-needle time to <120 minutes, target 4: reduce onset-to-puncture time to <200 minutes, target 5: reduce incidence of recurrent stroke by 10%, and target 6: reduce incidence of first-time stroke by 10%. QALY; quality adjusted life years, UK; United Kingdom.*

***Figure S3 – Population-level outcomes: incremental costs* and QALYs of reducing recurrent stroke by 10%***

| 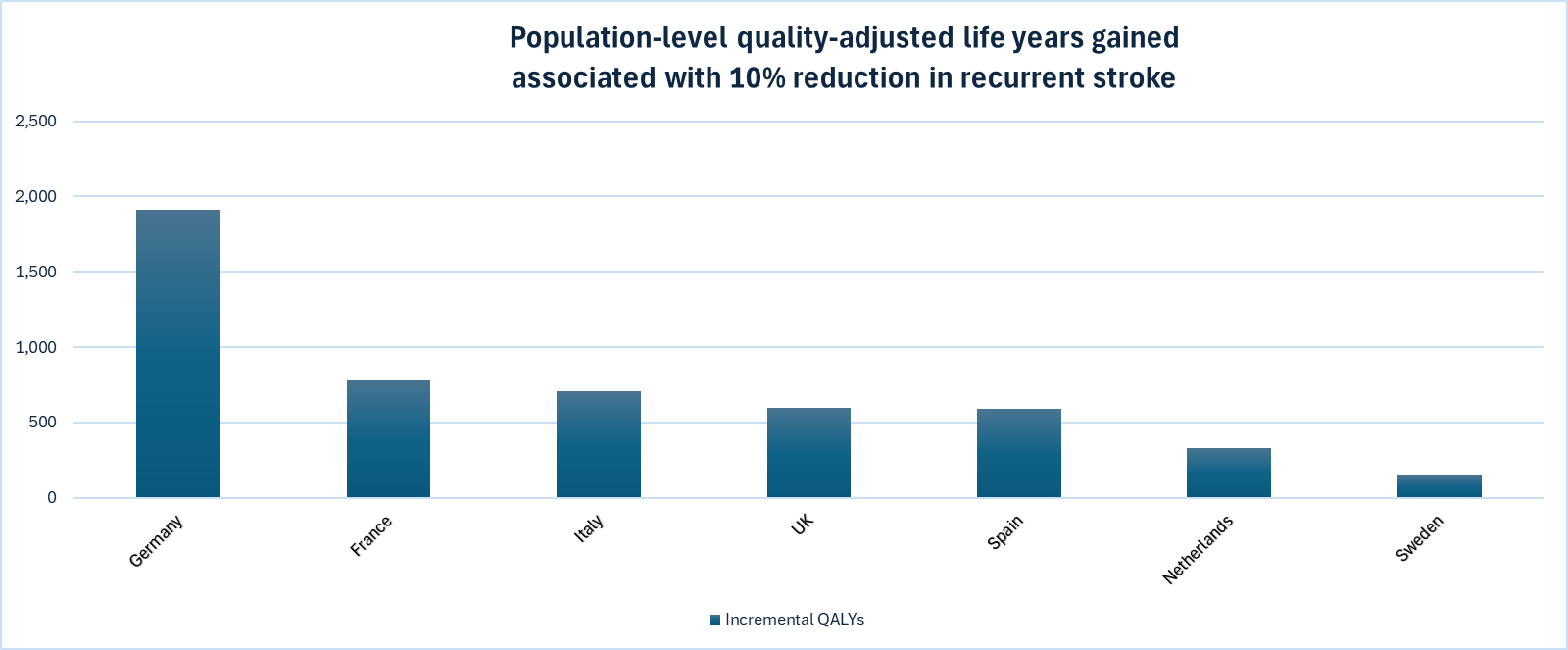 |
| --- |
| 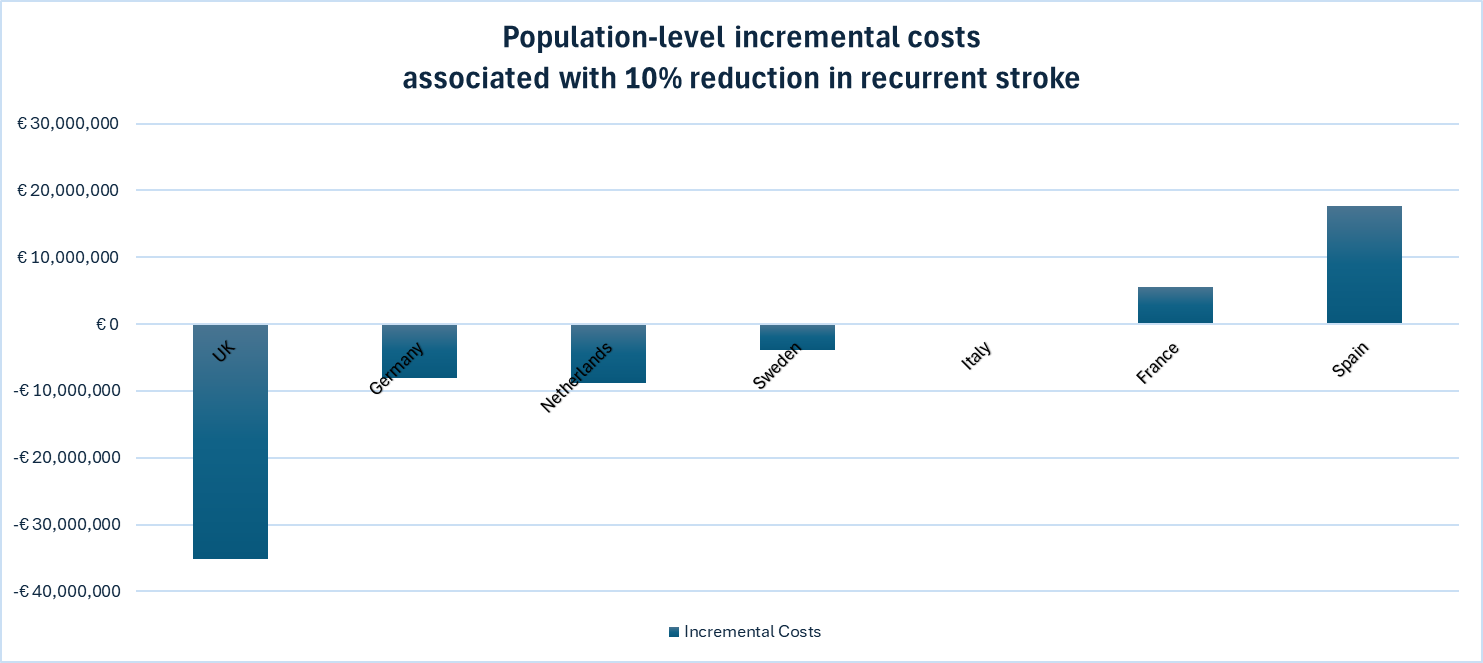 |

*** *Achieving the proposed targets may results in either positive or negative incremental costs, depending on the relative balance between long-term stroke care costs and first 90-day costs, including those associated with IVT and EVT, as well as cost differences linked to disability (mRS 3-5) and mortality (mRS 6) in respective countries; QALY; quality adjusted life years, UK; United Kingdom.*

Probabilistic results

Probabilistic sensitivity analysis demonstrated that primary prevention of AIS, modeled as a 10% reduction in first-time stroke incidence, was a robustly dominant strategy, leading to improved health outcomes at lower costs. In Spain, nearly all simulations confirmed dominance when achieving this target (Figure S4).

Probabilistic sensitivity analysis also demonstrated inherent parameter uncertainty in scenarios with small incremental QALYs and costs. Meeting some of the SAP-E targets resulted in only marginal gains in health outcomes and costs due to current care already being close to target levels. For example, in the Netherlands, increasing EVT rates from 4.6% to 5% (Target 2) and in the UK, increasing IVT rates from 11.7% to 15% (Target 1) produced only marginal incremental QALYs and costs (Figure S5). Hence, the probabilistic results could shift between dominance and non-dominance, and low and higher ICERs.

***Figure S4 – Probabilistic sensitivity analysis: Dominance cases***

| **Spain: Probabilistic results of meeting Target 6 (10% reduction in AIS incidence)**  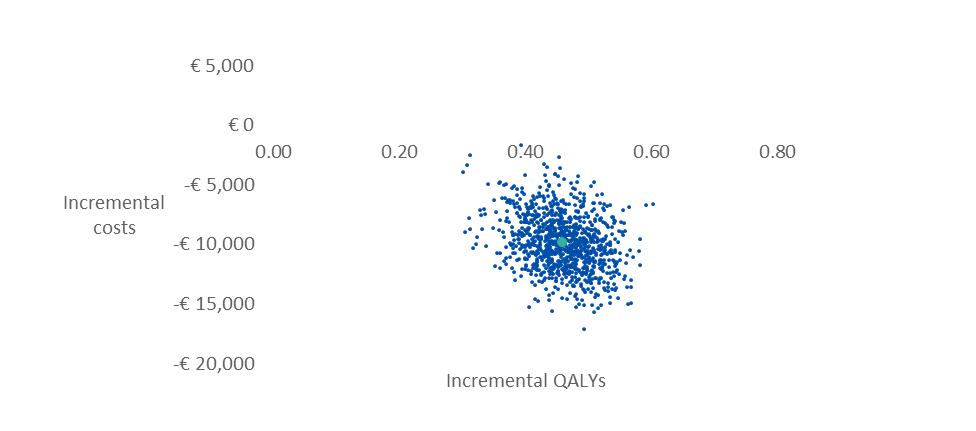 |
| --- |
| **Sweden: Probabilistic results of meeting Target 6 (10% reduction in AIS incidence)** |
| 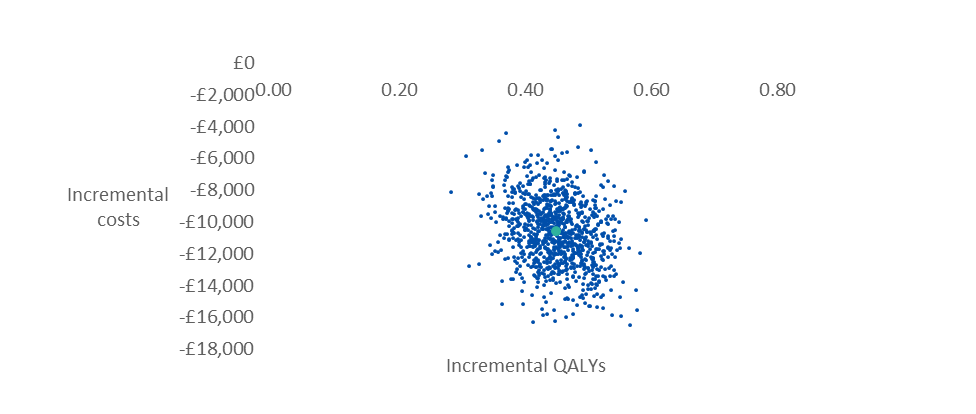 |
| **Italy: Probabilistic results of meeting Target 2 (increasing EVT rate to 5% from the current 1.7%)**  a. Cost-effectiveness plane |
| 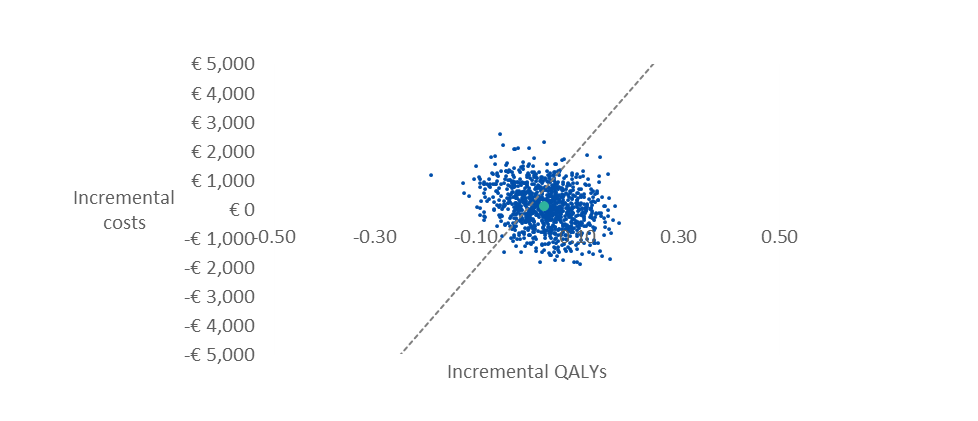  b. Cost-effectiveness acceptability curves  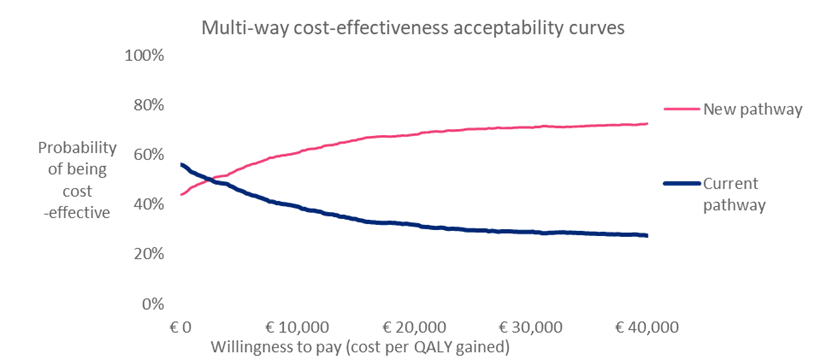 |

*Target 2: increase endovascular treatment rate to 5%. Target 6: reduce incidence of first-time stroke by 10%. AIS; acute ischemic stroke, EVT; endovascular treatment, QALY; quality adjusted life years.*

***Figure S5 – Probabilistic sensitivity analysis of meeting selected targets: non-dominance cases***

| **UK: Probabilistic results of meeting Target 1 (increasing IVT rate to 15% from the current 11.7%)**  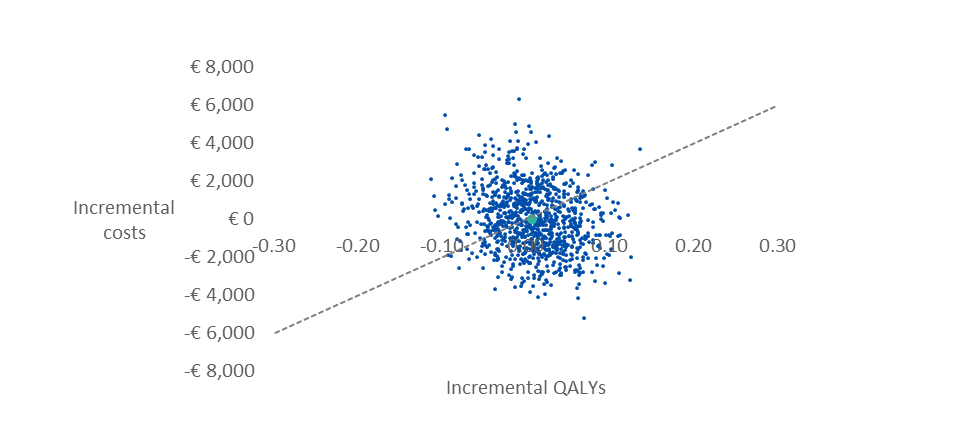 |
| --- |
| **The Netherlands: Probabilistic results of meeting Target 2 (increasing EVT rate to 5% from the current 4.6%)**  **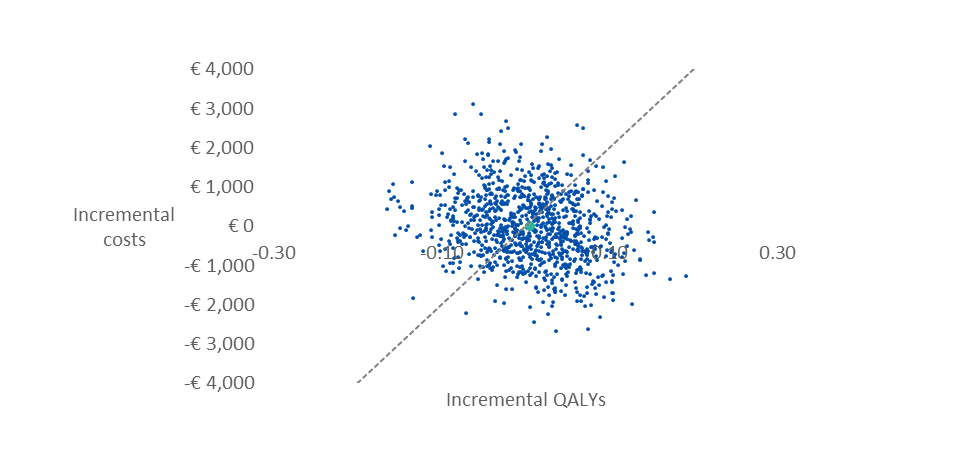** |
| **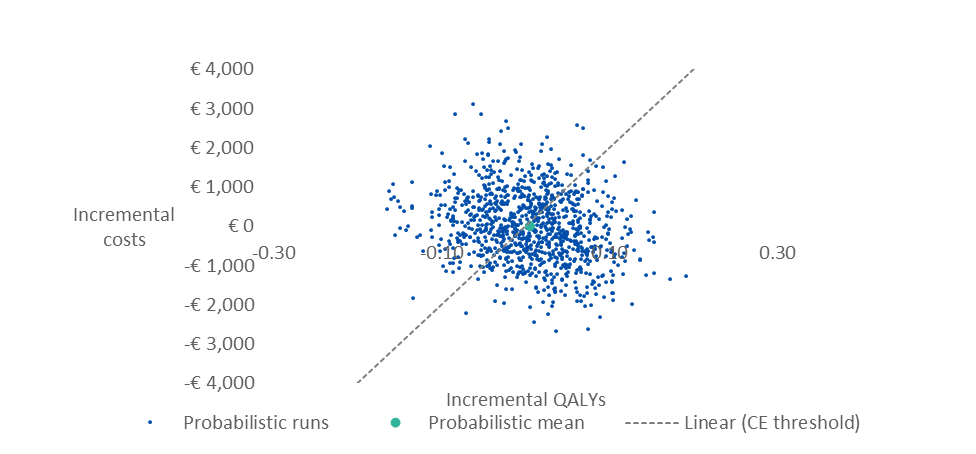** |

*Target 1: increase intravenous treatment rate to 15%. Target 2: increase endovascular treatment rate to 5. EVT; endovascular treatment, QALY; quality adjusted life years, UK; United Kingdom.*

1. **References**

1. VizHub - GBD Compare.

2. Hebant B, Guegan-Massardier E, Macaigne V, Triquenot-Bagan A. Ischemic stroke due to internal carotid artery dissection associated with an elongated styloid process (Eagle syndrome). J Neurol Sci. 2017 Jan 15;372:466–7.

3. Couture M, Finitsis S, Marnat G, Richard S, Bourcier R, Constant-Dits-Beaufils P, et al. Impact of Prior Antiplatelet Therapy on Outcomes After Endovascular Therapy for Acute Stroke: Endovascular Treatment in Ischemic Stroke Registry Results. Stroke. 2021 Dec;52(12):3864–72.

4. Aguiar de Sousa D, von Martial R, Abilleira S, Gattringer T, Kobayashi A, Gallofré M, et al. Access to and delivery of acute ischaemic stroke treatments: A survey of national scientific societies and stroke experts in 44 European countries. Eur Stroke J. 2019 Mar;4(1):13–28.

5. Moreu M, Scarica R, Pérez-García C, Rosati S, López-Frías A, Egido JA, et al. Mechanical thrombectomy is cost-effective versus medical management alone around Europe in patients with low ASPECTS. J Neurointerventional Surg. 2023 Jul;15(7):629–33.

6. WHO Global Health Observatory.

7. Haute Autorité de Santé. Choices in Methods for Economic Evaluation. Oct 2012 [Internet]. Available from: https://www.has-sante.fr/upload/docs/application/pdf/2012-10/choices_in_methods_for_economic_evaluation.pdf

8. Olié V, Grave C, Tuppin P, Duloquin G, Béjot Y, Gabet A. Patients Hospitalized for Ischemic Stroke and Intracerebral Hemorrhage in France: Time Trends (2008-2019), In-Hospital Outcomes, Age and Sex Differences. J Clin Med. 2022 Mar 17;11(6).

9. Federal Statistical Office. Annual consumer price index - France [Internet]. 2024. Available from: https://www.insee.fr/en/statistiques/serie/001765159

10. Rohde S, Weber W, Berlis A, Urbach H, Reimer P, Schramm P. Acute Endovascular Stroke Treatment in Germany in 2019 : Results from a Nationwide Database. Clin Neuroradiol. 2021 Mar;31(1):11–9.

11. Institute for Quality and Efficiency in Health Care. General Method. 2020;Version 6.0. Available from: https://www.iqwig.de/en/about-us/methods/

12. Alegiani AC, Dorn F, Herzberg M, Wollenweber FA, Kellert L, Siebert E, et al. Systematic evaluation of stroke thrombectomy in clinical practice: The German Stroke Registry Endovascular Treatment. Int J Stroke Off J Int Stroke Soc. 2019 Jun;14(4):372–80.

13. Statistisches Bundesamt. Consumer price index: Germany [Internet]. 2020. Available from: https://www-genesis.destatis.de/datenbank/online/statistic/61111/table/61111-0003/

14. Cappellari M, Bonetti B, Forlivesi S, Sajeva G, Naccarato M, Caruso P, et al. Acute revascularization treatments for ischemic stroke in the Stroke Units of Triveneto, northeast Italy: time to treatment and functional outcomes. J Thromb Thrombolysis. 2021 Jan;51(1):159–67.

15. AIFA. L’AIFA approva le nuove linee guida per la contrattazione dei prezzi e rimborsi dei farmaci. [cited 2025 Sep 17]; Available from: https://www.aifa.gov.it/-/l-aifa-approva-le-nuove-linee-guida-per-la-contrattazione-dei-prezzi-e-rimborsi-dei-farmaci

16. Sacco S, Stracci F, Cerone D, Ricci S, Carolei A. Epidemiology of stroke in Italy. Int J Stroke Off J Int Stroke Soc. 2011 Jun;6(3):219–27.

17. IstatData - CPI for Health. Harmonized index of consumer prices (base 2015=100) - annual average [Internet]. 2015. Available from: https://esploradati.istat.it/databrowser/#/en/dw/categories/IT1,Z0400PRI,1.0/PRI_HARCONEU

18. Compagne KCJ, Kappelhof M, Hinsenveld WH, Brouwer J, Goldhoorn RJB, Uyttenboogaart M, et al. Improvements in Endovascular Treatment for Acute Ischemic Stroke: A Longitudinal Study in the MR CLEAN Registry. Stroke. 2022 Jun;53(6):1863–72.

19. Zorginstituut Nederland. Richtlijn voor het uitvoeren van economische evaluaties in de gezondheidszorg. Herziene Versie 2024 [Internet]. Available from: https://www.zorginstituutnederland.nl/documenten/2024/01/16/richtlijn-voor-het-uitvoeren-van-economische-evaluaties-in-de-gezondheidszorg

20. Jansen IGH, Mulder MJHL, Goldhoorn RJB. Endovascular treatment for acute ischaemic stroke in routine clinical practice: prospective, observational cohort study (MR CLEAN Registry). BMJ. 2018 Mar 9;360:k949.

21. CBS Dutch Statistics Bureau. Consumer Price Index - The Netherlands [Internet]. CDID L528 DatasetID MM23; 2015. Available from: https://www.cbs.nl/en-gb/figures/detail/83131ENG

22. Requena M, Pérez de la Ossa N, Abilleira S, Cardona P, Urra X, Martí-Fabregas J, et al. Predictors of Endovascular Treatment Among Stroke Codes Activated Within 6 Hours From Symptom Onset. Stroke. 2018 Sep;49(9):2116–21.

23. Tejada Meza H, Lambea Gil Á, Villar Yus C, Pérez Lázaro C, Navarro Pérez MP, Campello Morer I, et al. [Three-month functional prognosis of patients hospitalised due to acute ischaemic stroke in Aragon: Rregional analysis of the impact of COVID-19]. Neurol Barc Spain. 2021 Sep;36(7):531–6.

24. de Andrés-Nogales F, Álvarez M, de Miquel MÁ, Segura T, Gil A, Cardona P, et al. Cost-effectiveness of mechanical thrombectomy using stent retriever after intravenous tissue plasminogen activator compared with intravenous tissue plasminogen activator alone in the treatment of acute ischaemic stroke due to large vessel occlusion in Spain. Eur Stroke J. 2017 Sep;2(3):272–84.

25. Amaya Pascasio L, Blanco Ruiz M, Milán Pinilla R, García Torrecillas JM, Arjona Padillo A, Del Toro Pérez C, et al. Stroke in Young Adults in Spain: Epidemiology and Risk Factors by Age. J Pers Med. 2023 Apr 29;13(5).

26. Instituto Nacional de Estadistica. National indices - Spain [Internet]. CDID ECOICOP preUnit health; Available from: https://www.ine.es/jaxiT3/Tabla.htm?t=50902

27. Mazya MV, Berglund A, Ahmed N, von Euler M, Holmin S, Laska AC, et al. Implementation of a Prehospital Stroke Triage System Using Symptom Severity and Teleconsultation in the Stockholm Stroke Triage Study. JAMA Neurol. 2020 Jun 1;77(6):691–9.

28. LFNAR. General guidelines for economic evaluations from the Pharmaceutical Benefits Board. 2003:2 [Internet]. Available from: https://www.tlv.se/download/18.2e53241415e842ce95514e9/1510316396792/Guidelines-for-economic-evaluations-LFNAR-2003-2.pdf

29. Darehed D, Blom M, Glader EL, Niklasson J, Norrving B, Eriksson M. Time Trends and Monthly Variation in Swedish Acute Stroke Care. Front Neurol. 2019;10:1177.

30. Statistikmyndigheten. Statistics Sweden, CPI, Indices for Main Groups, annual averages [Internet]. 1980. Available from: Statistics Sweden, CPI, Indices for Main Groups, annual averages

31. Gill S. Reducing door to needle time for stroke thrombolysis. BMJ Qual Improv Rep. 2014;3(1).

32. Dhillon PS, Soo E, Butt W, Nguyen TN, Barrett E, Podlasek A, et al. Comparison Between In‐Hospital and Community‐Onset Stroke Treated With Endovascular Thrombectomy: A Propensity Score–Matched Cohort Study. Stroke Vasc Interv Neurol. 2023 Jul 1;3(4):e000816.

33. York Health Economics Consortium. Discount Rate [online]. 2016 [Internet]. Available from: https://yhec.co.uk/glossary/discount-rate/

34. National Institute for Health and Care Excellence (NICE). Stroke and transient ischaemic attack in over 16s: diagnosis and initial management. Vol. 2022 Apr 13. London; 2022.

35. Jones K, Weatherly H, Birch S, Castelli A. Unit Costs of Health and Social Care [Internet]. 2024. Available from: https://discovery.ucl.ac.uk/id/eprint/10211930/
